# Antibody-drug conjugates—an emerging class of cancer treatment

**DOI:** 10.1038/bjc.2015.435

**Published:** 2016-01-07

**Authors:** Nikolaos Diamantis, Udai Banerji

**Affiliations:** 1 Drug Development Unit, The Institute of Cancer Research and The Royal Marsden, Downs Road, Sutton, London SM2 5PT, UK

**Keywords:** antibody-drug conjugate, target antigen, cytotoxic payload, linker, T-DM1, brentuximab vedotin, MMAE, resistance

## Abstract

Antibody-drug conjugates (ADCs) are an emerging novel class of anticancer treatment agents that combines the selectivity of targeted treatment with the cytotoxic potency of chemotherapy drugs. New linker technology associated with novel highly potent cytotoxic payloads has permitted the development of more effective and safe ADCs. In recent years, two ADCs have been licensed, T-DM1 and brentuximab vedotin, and are already establishing their place in cancer treatment. A plethora of ADCs are being investigated in phases I and II trials, emerging data of which appears promising. As we deepen our understanding of what makes a successful ADC, an increasing number of ADCs will likely become viable treatment options as single agents or in combination with chemotherapy. This review will present the philosophy underlying ADCs, their main characteristics and current research developments with a focus on ADCs in solid tumours.

Paul Ehrlich's vision of a rational targeted strategy against invading microbes or malignant cells has been a driving research throughout the past century, bringing a scientific and therapeutic revolution in cancer treatment. Although antibody-drug conjugates (ADCs) have been under investigation for decades, it is only recently with the advent of significant advances in engineering new linker and conjugation technologies together with highly potent cytotoxic drugs that we have realised the ADC's true potential (Panowksi *et al*, 2014). Monoclonal antibodies have proved to have an important role in cancer treatment with drugs such as trastuzumab, pertuzumab, cetuximab and rituximab becoming the standard of care in selected solid tumours and lymphomas. Classic chemotherapy, the mainstay of anticancer treatment, demonstrates limited selectivity against cancer cells leading to a small therapeutic window, thus limiting its efficacy.

Antibody-drug conjugates could bring these two classes of drugs with their complementing properties together, in creating a highly selective and highly cytotoxic cancer treatment with an increased therapeutic window, as envisaged by Paul Ehrlich (Hughes, 2010). Looking back into the reasons for previous failures gives us an opportunity to identify the crucial characteristics that will make ADC an effective anticancer treatment. The low chemotherapy drug potency, unstable linkers and low antigen selectivity are the most commonly identified weaknesses limiting the efficacy of an ADC (Perez *et al*, 2014). In addition, in some of the first clinical trials murine antibodies were used leading to high rates of immunogenicity and, therefore, to low efficacy (Hughes, 2010).

Antibody-drug conjugates have a complex structure with many moving parts, each of which has different properties and desirable characteristics. An ADC can be divided into three main structural units: the antibody, the cytotoxic agent and the linker, which will be discussed below.

## Antibody-drug conjugates

### Target antigen

The ideal target antigen should be: (a) highly expressed with limited heterogeneity across the tumour and with low normal tissue expression, for example, T-DM1 targets HER-2, a receptor that reaches levels of expression of 2 × 10^6^ on HER-2-positive cancer cells compared with 2 × 10^4^ on other cells (Shefet-Carasso and Benhar, 2015); (b) there should be minimal antigen shedding to prevent the antibody binding to its target within the circulation; and (c) the antibody should be well internalised by receptor-mediated endocytosis and should not be modulated during endocytosis. The target antigen should not be downregulated after treatment with the ADC (Mack *et al*, 2014). The minimum threshold of the different variables that are required to a make a tumour antigen an effective target is still undetermined and interdependent. Studies in lymphoma and prostate cancer have shown that a minimum value of tumour-antigen density is a prerequisite for ADC efficacy. The biomarker analysis study for TDM1 showed that although it was active across different HER-2 expression subgroups, patients with tumours who expressed HER-2 more highly derived the greatest benefit (Baselga *et al*, 2013). The desirable cutoff value of antigen expression varies greatly and depends on other target antigen properties such as the internalisation rate and binding affinity, as well as other ADC characteristics, such as cytotoxic payload and linker stability. There is evidence that ADCs can be effective even when they target antigens with low expression given minimal normal tissue expression (Perez *et al*, 2014). The problem of non-homogeneous expression of the target antigen in solid tumours could potentially be addressed by the bystander effect, that is, the process by which membrane-permeable free cytotoxic payload is able to induce cell death to the neighbouring cells after being internalised and cleaved from the linker. Conversely, the bystander effect can increase the ADCs off-target systemic toxicity. Various factors can affect the rate of internalisation of the ADC in the cancer cell, which is a poorly understood process. One such factor is the epitope on the target antigen. For example, different epitopes of the HER-2 receptor have resulted in significantly different rates of internalisation and degradation of the mAb-Ag molecule. In addition, there are other difficulties while targeting cancer cell surface antigens, such as the high interstitial tumour pressure, downregulation of the antigen and the presence of other physical and kinetic barriers that diminish the cytotoxic payload uptake (Mack *et al*, 2014; Perez *et al*, 2014). Although most licensed or at an advanced research stage ADCs target tumour antigens, alternative approaches are actively being investigated to overcome some of the above mentioned limitations (Casi and Neri, 2012).

**Figure 1:**
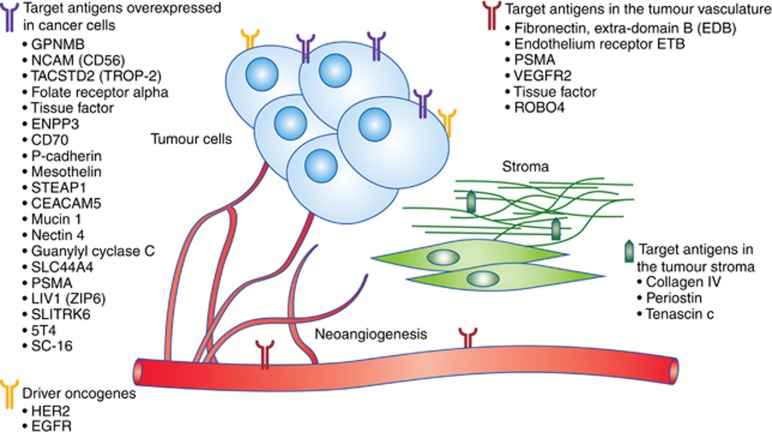
**Target antigens for ADCs in solid tumours.**

Another approach is to target antigens in stroma and vasculature. There is evidence in the preclinical and clinical setting that components of the neovasculature subendothelial extracellular matrix and of the tumour stroma could be valuable target antigens. A characteristic example is the extra-domain B (ED-B) of fibronectin which is a marker of angiogenesis, specifically highly expressed in vasculature of aggressive solid tumours (Palumbo *et al*, 2011) (Figure 1). The human mAb L19 which targets the ED-B has been combined with different effector molecules and has been studied extensively as a potential ADC. This target was successfully exploited by conjugating it with tubulin inhibitors showing that non-internalising vascular targeting ADCs could offer other treatment approaches (Perrino *et al*, 2014), (Table 1).

A further interesting approach to targeting antigens is to target the tumour-initiating cells or cancer stem cells (TICs). The hypothesis is that since TICs comprise an aggressive sub-population of tumour cells that are responsible for tumour growth, metastasis and recurrence, targeting these cells will have a big impact on the disease control (Visvader and Lindeman, 2008). Sapra *et al* (2013) have developed an ADC combined with a tubulin inhibitor that targets 5T4 oncofetal antigen, which is an antigen expressed in TICs in NSCLC and has been associated with an invasive phenotype. A phase I trial investigating this ADC is currently recruiting patients with NSCLC and other solid tumours (Figure 1).

### Antibody selection

Antibody engineering has made breakthroughs over recent years making it feasible to produce humanised and fully human antibodies as the basic components of ADCs. The early generation ADCs used murine antibodies causing significant immunogenicity, with many patients producing human anti-mouse antibodies thus reducing the efficacy of treatment.

The most commonly used antibody format currently is human IgG isotypes and in particular IgG1 (Hughes, 2010; Perez *et al*, 2014). The antibodies once part of the ADC can retain their original properties and activate immune functions such as antibody-dependent cellular cytotoxicity (ADCC) or complement-dependent cytotoxicity (Perez *et al*, 2014). They could still act as receptor inhibitors or signal modulators. One example of complementary action is T-DM1 whose Ab's retain effector functions have been shown to activate ADCC (Junttila *et al*, 2011). The independent function of the antibody is not always beneficial and complementary to ADC efficacy, especially when Ab binding is sufficient to produce a cytotoxic effect. The Ab's independent effector functions could lead to increased toxicity, reduced tumour localisation and internalisation of the ADC (Sapra and Shor, 2013).

### Linkers and conjugation

The linker plays a crucial role since its properties greatly influence the ADC's pharmacokinetics, therapeutic index and efficacy (Hughes, 2010; Flygare *et al*, 2013; Shefet-Carasso and Benhar, 2015). The ideal linker should be stable so that the ADC does not release the cytotoxic drug before reaching its target and causing off-target toxicity. At the same time it should be able to release the drug efficiently once internalised (Teicher and Chari, 2011).

Another important factor is how many of the drug molecules will be loaded onto the antibody: the drug-antibody ratio (DAR). Attaching too few of the drug molecules will lead to decreased efficacy. Attach too many and the ADC will become unstable with altered pharmacokinetic properties, increased plasma clearance, reduced half-life and increased systemic toxicity (Perez *et al*, 2014). The currently licensed ADCs with proven activity are produced by nonspecific conjugation to lysine residues and to some degree consist of an undesirable heterogeneous mixture of ADCs containing drug molecules with high DAR. There are ongoing efforts for more homogeneous ADCs with increased number of drug molecules stably linked to the Ab. The optimal DAR is undetermined and highly dependent on other ADC variables; however, more commonly the ADCs aim to attain a DAR close to 4. (Hamblett *et al*, 2004; Teicher and Chari, 2011). Site-specific conjugation, has been an important step in ADC development, enabled the production of homogeneous ADCs with the desired and pre-specified DAR by using techniques such as engineered cysteine residue, non-canonical amino acid incorporation or modification of peptide tags (Panowksi *et al*, 2014; Agarwal and Bertozzi, 2015).

Linkers can be divided in non-cleavable and cleavable. Non-cleavable linkers have the characteristic that following ADC lysosomal degradation the cytotoxic payload remains active while still being attached to the linker and an amino acid residue. T-DM1 is an ADC that uses a non-cleavable linker (Junttila *et al*, 2011; Shefet-Carasso and Benhar, 2015).

Cleavable linkers use different methods to release cytotoxic drug, increasing the possibility of the bystander effect. There are acid-sensitive linkers that will release the free drug in the low pH conditions in lysosomes or endosomes. Such a mechanism was used by gemtuzumab ozogamicin and its linker was characterised by certain plasma instability (Hamann *et al*, 2002; Shefet-Carasso and Benhar, 2015). Inotuzumab ozogamicin, now in phase II trials, uses the same linker and it is more stable (Boghaert *et al*, 2008). Another type of cleavable linker are lysosomal protease-sensitive linkers with the licensed example of brentuximab vedotin (Senter and Sievers, 2012). A third type are glutathione-sensitive linkers that benefit from the higher concentration of glutathione in tumour cells. (Sapra *et al*, 2011). Each approach has different advantages and disadvantages, so the choice of the linker can only be determined by taking into account all the other components of the ADC.

### Cytotoxic payload

To create an effective ADC, it is imperative to have a potent cytotoxic payload. The first generation of ADCs used classical chemotherapy drugs such as doxorubicin and methotrexate with the benefit of a well-known toxicity profile (Shefet-Carasso and Benhar, 2015). Repeated studies, however, have shown that the actual concentration of the cytotoxic payload in tumour cells is minimal with only 1–2% of the administered dose reaching the tumour (Teicher and Chari, 2011). It is evident that the optimal chemotherapy drug used should be extremely potent, being effective at picomolar or nanomolar concentrations.

There are two main categories of cytotoxic drugs used in ADC development: microtubule inhibitors and DNA-damaging drugs. Auristatins block tubulin assembly and cause G2/M phase cell cycle arrest; they are the most commonly used payloads, accounting for a majority of cytotoxic payloads used in ADCs currently investigated (Sapra and Shor, 2013; Bouchard *et al*, 2014). Monomethyl auristatin E, an auristatin derivative (MMAE) is the cytotoxic payload of brentuximab vendotin and has a free drug IC_50_: 10^−11^–10^−9^ allowing it to be effective in the low nanomolar range (Gerber *et al*, 2009). Maytansinoids, another class of tubulin inhibitors, have also been used successfully in the ADC development. The cytotoxic payload of trastuzumab emtansine (T-DM1) DM1 is a highly potent maytansinoid, developed by Immunogen, with a free drug IC_50_: 10^−11^–10^−9^. Another maytansinoid drug, DM4 incorporated on SAR3419, is being investigated in phase II trials exhibiting significant ‘bystander effect' as the ADC's metabolites have lipophilic properties allowing crossing of the cellular membrane (Blanc *et al*, 2011). Tubulysins are a promising new class of tubulin inhibitors. Tubulisin analogues were successfully conjugated to trastuzumab forming a stable and potent ADC (Cohen *et al*, 2014). The DNA-damaging agents have the ability to be active throughout the different cell cycle phases. Duocarmycin is a powerful cytotoxic alkylating compound that binds to the minor groove of DNA and has shown activity against various multidrug-resistant models. Duocarmycin-based ADCs are currently under investigation in a phase 1 trial setting conjugated to the anti-HER-2 antibody, trastuzumab (Van Herpen *et al*, 2015).

Calicheamicin is a potent antitumour antibiotic that causes double-strand DNA breaks and rapid cell death by binding to the DNA's minor groove. It is less dependent on cell cycle progression making it potentially useful against TICs who have lower rates of proliferation (Gupta *et al*, 2009; Sapra *et al*, 2011). Gemtuzumab ozogamicin and other ADCs such as inotuzumab ozogamicin in non-Hodgkin lymphoma and MDX-1203 in renal cancer are using these agents (Owonikoko *et al*, 2014).

A potential new drug under investigation is α-amanitin, an RNA polymerase II inhibitor in the picomolar range, derived from the mushroom, *Amanita phalloide*s (Moldenhauer *et al*, 2012). Other topoisomerase inhibitors under investigation is the SN38, the active metabolite of irinotecan (Palakurthi, 2015). Another new category is the pyrrolobenzodiazepines (PBDs) that bind to discrete DNA sequences causing lethal lesions and have interestingly not been found to have cross-resistance with common chemotherapeutic agents (Bouchard *et al*, 2014).

A different approach, but in line with ADC principles, are the radioimmunoconjugates where radionuclides are the cytotoxic payloads linked to monoclonal antibodies. Two radioimmunoconjugates targeting CD20 have been approved for treatment of refractory HodHL, (Yttrium-90-ibritumomab tiuxetan and iodine-131-tositumomab). Despite its efficacy due to under-utilisation tositumomab manufacturer has discontinued its production. In solid tumours, radioimmunoconjugates are being investigated, particularly in treating minimal residual disease in prostate and colorectal cancer among others (Kraeber-Bodere *et al*, 2014).

## Clinical experience

### T-DM1, Kadcyla

The only ADC licensed in non-haematological malignancies is ado-trastuzumab-emtansine (T-DM1, Kadcyla, Roche, Genentech), the antibody used is the well-known trastuzumab, a humanised IgG1 anti-HER-2 Ab linked with a stable non-cleavable linker to the maytansinoid DM1 (LoRusso *et al*, 2011; Verma *et al*, 2012). T-DM1 has been approved in the second line setting by the FDA in 2013 for HER-2-positive patients who had previously received treatment with trastuzumab and taxane chemotherapy. T-DM1 has an overall tolerable toxicity profile with most common adverse events being fatigue, transaminitis, nausea, thrombocytopenia and rash. In USA, T-DM1 carries black box warnings for hepatotoxicity, embryo-fetal and cardiac toxicity *P*<0.001) (Verma *et al*, 2012).

T-DM1 is also being investigated as a single agent comparted to docetaxel in previously treated gastric cancer with results expected in 2015 (GATSBY trial, NCT01641939) (Shah, 2015).

### Brentuximab vedotin, Adcetris

Brentuximab vedotin (BV, Adcetris, Seattle Genetics) is composed of an anti-CD30 mAb connected with a cleavable peptide to the highly potent tubulin inhibitor MMAE discussed previously. CD30 is a member of the tumour necrosis factor (TNF) family identified on Reed–Sternberg cells of classical Hodgkin lymphoma (HL). Binding of BV to the cell surface will lead to internalisation and lysosomal proteolytic cleavage of the linker releasing the MMAE (Senter and Sievers, 2012; Sievers and Senter, 2013).

BV has gained approval for the treatment of patients with relapsed or refractory CD30+ HL following autologous stem cell transplant (ASCT) or patients not legible for ASCT who have failed at least two other chemotherapy treatments. BV has also been approved for patients with anaplastic large cell lymphoma (ALCL) as a second line. The accelerated approval for Hodgkin's lymphoma was based on a single-arm phase II clinical trial, where there was a 73% response rate, 32% complete remission and a median duration 20.5 months (Younes *et al*, 2012). The indication for ALCL was established based on the impressive results of the phase II study. Patients at this study had an 86% overall response rate and 54% complete responses (Pro *et al*, 2012). The most common adverse reactions were peripheral sensory neuropathy, neutropenia, fatigue, nausea and thrombocytopenia. In USA, BV carries a black box warning for progressive multifocal leukoencephalopathy (Younes *et al*, 2012).

### Gemtuzumab ozogamicin, Mylotarg

Gemtuzumab ozoogamicin (GO, Mylotarg, Pfizer) was the first ADC to be approved by the FDA in 2000. It was licensed as a monotherapy in patients over the age of 60 with acute myelogenous leukemia (AML) who were not candidates for cytotoxic chemotherapy. GO consisted of a humanised IgG4 mAb directed against CD33 a surface antigen present in 85–90% of AML linked to a calicheamicin cytotoxin with positive results coming from the first single-agent phase II studies achieving 30% remissions (Tsimberidou *et al*, 2006). In 2010, the results of a post-approval phase III study showed no clinical benefit. In fact, the patients in the GO arm had a higher risk of fatal AEs and this trial led to the retraction of its FDA licence approval (Petersdorf *et al*, 2013). The failures were attributed to an unstable linker allowing premature release of the cytotoxic payload and to the not-so-selective antibody target (ten Cate *et al*, 2009).

More recent trials such as the ALFA-0701 using intermittent dosing regimens have showed good results, improving event-free survival and overall survival in patients with AML, reigniting discussions about the future of GO (Castaigne *et al*, 2012).

### Clinical trial setting

Research in ADCs has been very active with almost 120 total clinical trials with the majority focusing on haematological malignancies. Interest in solid tumours is evident with 52 open phase I/II studies There are approximately 50 unique ADCs under investigation, with 35 ADCs investigated in solid tumours (Mullard, 2013).

### Putative mechanisms of resistance

Cancer cells under the pressure of any treatment develop resistance mechanisms that will allow them to survive. These mechanisms are complex and variable confronting the ADC in each of its different components, the cytotoxic drug, the monoclonal antibody or by activating survival signalling pathways (Shefet-Carasso and Benhar, 2015).

The cytotoxic payload of the ADC is subject to the same multidrug resistance mechanisms as conventional chemotherapy drugs. Some of the most commonly used drugs in the new generation ADCs such as auristatins (MMAE) and calicheamicin are substrates of P-glycoprotein. Resistance can also emerge from mechanisms that will limit the intracellular concentration of the ADC. Barok *et al* (2014) have described such mechanisms in their work with T-DM1, that is, downregulation of the target antigen, reduced internalisation of the ADC, diminished lysosomal degradation or increased ADC recycling to the cell surface and masking of the antigen epitope. There is also evidence that activation of the PI3K/AKT, MEK/ERK and JAK/STAT pathways leads to increased ADC resistance indicating that a potential way ahead could be combinations with small molecule inhibitors (Shefet-Carasso and Benhar, 2015). Clonal expansion of resistant cells and intratumoural heterogeneity representing populations of cells that are resistant *de novo* within the same tumour, are likely to influence resistance to ADCs (Gerlinger *et al*, 2012).

## Conclusion: future prospects

Predictive biomarkers are essential to ensure we are offering the most effective treatments to the group of patients most likely to benefit from them. Development of specific tests that will determine the chances of responding to treatment to an ADC by investigating the level of expression of the target antigen similar to the immunohistochemical (IHC) confirmation of HER-2 expression are already being explored (Mack *et al*, 2014). Efforts are ongoing in order to move away from IHC-based techniques that require tissue biopsy and use circulating tumour cells or imaging techniques to identify the patient population more likely to respond (Mack *et al*, 2014).

Another approach to ADCs is to use high-affinity molecules like folic acid or growth hormones as cytotoxic payload carriers or small antibody fragments such as diabodies or minibodies. The rationale behind this approach is that it might result in more effective drug delivery and increased antitumour activity. However, this approach will also limit the pharmacokinetic benefits of an ADC resulting in more rapid clearance and larger volume of distribution.

Combination strategies are actively explored in many ongoing clinical trials. T-DM1 is being investigated in combination with pertuzumab (Phillips *et al*, 2014). Other combinations of ADCs investigated are with conventional chemotherapies, for example, brentuximab with modified AVD, PI3K inhibitors like BYL719 with T-DM1 or with TKI inhibitors like neratinib.

There remain major hurdles that ADCs need to overcome: low delivery efficiency, target antigens expressed in normal tissues, the heterogeneity of target antigen expression in the tumour and more (Teicher and Chari, 2011). The future of ADCs seems promising as the combination of new linker technologies and more powerful cytotoxic payloads leads to the emergence of more stable and effective ADCs.

## Figures and Tables

**Table 1:** Target antigens in solid tumours under research

**Name**	**ADC**	**Lead indication**
**Target antigens overexpressed in cancer cells**		
GPNMB	Glembatumumab vedotin [1]	Breast cancer and melanoma
CD56	Lorvotuzumab mertansine (IMGN-901) [2]	SCLC
TACSTD2 (TROP2)	sacituzumab govitecan (IMMU-132) [3]	TNBC and pancreatic cancer
CEACAM5	Labetuzumab SN-38 [4]	Colorectal cancer
Folate receptor-α	• Mirvetuximab soravtansine (IMGN-853) [5] • Vintafolide [6]	Ovarian and endometrial cancer
Mucin 1 (Sialoglycotope CA6)	SAR-566658 [7]	Breast, ovarian, cervical, lung and pancreatic cancer
STEAP1	Vandortuzumab vedotin RG-7450 [8]	Prostate cancer
Mesothelin	• DMOT4039A [9] • Anetumab ravtensine (BAY-94–9343) [10]	Ovarian, pancreatic cancer and mesothelioma
Nectin 4	• Enfortumab vedotin (ASG-22M6E) [11] • ASC-22CE [12]	Bladder, breast, lung and pancreatic cancer
ENPP3	AGS-16M8F [13]	Renal cell carcinoma, liver carcinoma and prostate cancer
Guanylyl cyclase C (GCC)	Indusatumab vedotin (MLN-0264) [14]	Pancreatic and colorectal cancer
SLC44A4	ASG-5ME [15]	Pancreatic, gastric and prostate cancer
*NaPi2b*	• anti-NaPi2b ADC, Lifastuzumab vedotin [16]	Non-small cell lung cancer and platinum-resistant ovarian cancer
CD70 *(TNFSF7)*	• DNIB0600A [17]• AMG-172 [18] • MDX-1203 [19] • Vorsetuzumab mafodotin SGN-75 [20]	Renal cell carcinoma
CA9, Carbonic anhydrase	BAY79–4620 [21]	Solid tumours
5T4 (TPBG)	PF 06263507 [22]	Solid tumours
SLTRK6	ASG-15ME [23]	Bladder cancer
SC-16 *(anti-Fyn3)*	SC16LD6.5 [24]	SCLC
		NSCLC and ovarian cancer
Tissue factor	HuMax-TF-ADC (TF-011-MMAE) [25]	Solid tumours
LIV-1 (ZIP6)	SGN-LIV1A [26]	Breast cancer
P-Cadherin	PCA062 [27]	Solid tumours
PSMA	• MLN2704 [28] • PSMA-ADC [29]	Prostate cancer
**Target antigens in the tumour vasculature and stroma**		
Fibronectin Extra-domain B (ED-B)	Human mAb L19 and F8 [30]	Solid tumours
Endothelin receptor ETB	RG-7636 [31]	Melanoma
VEGFR2 (CD309)	Anti-VEGFR-2ScFv-As2O3-stealth Nanoparticles [32]	Solid tumours
Tenascin c	Anti-TnC-A1 antibody SIP(F16) [33]	Solid tumours
Collagen IV	Cytotoxic immunoconjugates [34]	Solid tumours
Periostin	Anti-periostin antibody [35]	Solid tumours
**Target antigens regulated from driver oncogenes**		
HER 2	• T-DM1[36] • ARX788 [37] • SYD985 [38]	Breast cancer
EGFR	• ABT-414 [39] • IMGN289 [40] • AMG-595 [41]	Glioblastoma, NSCLC, head and neck, breast, oesophageal
**Target antigens in haematological malignancies**		
CD30	• Brentuximab vedotin [42] • Iratumumab MDX-060 [43]	HL and ALCL
CD22	• Inotuzumab ozogamicin (CMC-544) [44] • Pinatuzumab vedotin [45] • Epratuzumab SN38 [46]	NHL and ALL
CD79b	• Polatuzumab vedotin [47]	DLBCL and follicular NHL
CD19	• Coltuximab ravtansine [48] • SAR-3419 [49]	DLBCL and ALL
CD138	Indatuximab ravtansine [50]	Multiple myeloma
CD74	Milatuzumab doxorubicin [51]	CLL, NHL and multiple myeloma
CD37	IMGN-529 [52]	NHL and CLL
CD33	• IMGN779 [53] • SGN CD33 A [54]	AML
CD19	SGN-CD19A [55]	ALL and NHL,
CD98	IGN523 [56]	AML

Abbreviations: ADC=antibody-drug conjugate; ALL=acute lymphocytic leukemia; AML=acute myelogenous leukemia; CLL=chronic lymphocytic leukemia; DLBCL=diffuse large B-cell lymphoma; HL=Hodgkin lymphoma; NHL=non-Hodgkin lymphoma; NSCLC=non-small cell lung cancer; SCLC=small cell lung cancer; TNBC=triple-negative breast cancer.

Please refer to the Supplementary Data File for all references cited in this table.
