# Supplementary material for: Antibody-drug conjugates—an emerging class of cancer treatment
Source: Br J Cancer. 2016 Jan 7;114(4):362–7. doi: 10.1038/bjc.2015.435 (PMC4815767; doi:10.1038/bjc.2015.435)
Supplement: Supplementary Data [file bjc2015435x1.doc]

**Supplementary Data: references cited in Table 1**

1. Yardley, D.A., et al., EMERGE: A Randomized Phase II Study of the Antibody-Drug Conjugate Glembatumumab Vedotin in Advanced Glycoprotein NMB-Expressing Breast Cancer. J Clin Oncol, 2015. **33**(14): p. 1609-19.

2. Berdeja, J.G., Lorvotuzumab mertansine: antibody-drug-conjugate for CD56+ multiple myeloma. Front Biosci (Landmark Ed), 2014. **19**: p. 163-70.

3. Starodub, A.N., et al., First-in-Human Trial of a Novel Anti-Trop-2 Antibody-SN-38 Conjugate, Sacituzumab Govitecan, for the Treatment of Diverse Metastatic Solid Tumors. Clin Cancer Res, 2015. **21**(17): p. 3870-8.

4. Govindan, S.V., et al., CEACAM5-targeted therapy of human colonic and pancreatic cancer xenografts with potent labetuzumab-SN-38 immunoconjugates. Clin Cancer Res, 2009. **15**(19): p. 6052-61.

5. Ab, O., et al., IMGN853, a Folate Receptor-alpha (FRalpha)-Targeting Antibody-Drug Conjugate, Exhibits Potent Targeted Antitumor Activity against FRalpha-Expressing Tumors. Mol Cancer Ther, 2015. **14**(7): p. 1605-13.

6. Naumann, R.W., et al., PRECEDENT: a randomized phase II trial comparing vintafolide (EC145) and pegylated liposomal doxorubicin (PLD) in combination versus PLD alone in patients with platinum-resistant ovarian cancer. J Clin Oncol, 2013. **31**(35): p. 4400-6.

7. Valentina Boni1, O.R., Drew Rasco3, Carlos Gomez-Roca4, Emiliano Calvo1, John C. Morris2, Anthony W. Tolcher3, Sylvie Assadourian5, Helene Guillemin5, and Jean-Pierre Delord4, Abstract A73: A Phase I first-in-human (FIH) study of SAR566658, an anti CA6-antibody drug conjugate (ADC), in patients (Pts) with CA6-positive advanced solid tumors (STs) (NCT01156870). Mol Cancer Ther November 2013 12; A73, 2013.

8. Study of 89Zr-DFO-MSTP2109A in Patients With Prostate Cancer. NCT01774071, 2015.

9. Colin D. Weekes, L.E.L., Mitesh J. Borad, Johannes Voortman, Robert R. McWilliams, Jennifer Robinson Diamond, Elisabeth De Vries, Henk M.W. Verheul, Christopher Hanyoung Lieu, Huibin Yue, Yulei Wang, Suzie Scales, Divya Samineni, Katie Wood, Flavia Brunstein, Daniel J. Maslyar, George P. Kim, A phase I study of DMOT4039A, an antibody-drug conjugate (ADC) targeting mesothelin (MSLN), in patients (pts) with unresectable pancreatic (PC) or platinum-resistant ovarian cancer (OC). J Clin Oncol 32:5s, 2014 (suppl; abstr 2529) 2014.

10. Golfier, S., et al., Anetumab ravtansine: a novel mesothelin-targeting antibody-drug conjugate cures tumors with heterogeneous target expression favored by bystander effect. Mol Cancer Ther, 2014. **13**(6): p. 1537-48.

11. A Study of the Safety and Pharmacokinetics of AGS-22M6E in Subjects With Malignant Solid Tumors That Express Nectin-4. NCT01409135, 2015.

12. A Study of Escalating Doses of ASG-22CE Given as Monotherapy in Subjects With Metastatic Urothelial Cancer and Other Malignant Solid Tumors That Express Nectin-4. NCT02091999, 2015.

13. J. M. Gudas, M.T., Z. An, X. C. Jia, K. J. Morrison, R. K. Morrison, M. Vincent, P. Yang, S. B. Kanner, A. Jakobovits, Use of AGS-16M8F as a novel antibody drug conjugate (ADC) for treating renal cancers. J Clin Oncol 28, 2010 (suppl; abstr e15014), 2010.

14. A Study of MLN0264 in Patients With Pancreatic Cancer. NCT02202785, 2015.

15. Andrew L. Coveler, D.D.V.H., Andrew H. Ko, Nancy Cherry Whiting, Baiteng Zhao, Brian M. Wolpin, A phase I study of ASG-5ME, a novel antibody-drug conjugate, in pancreatic ductal adenocarcinoma. J Clin Oncol 31, 2013 (suppl 4; abstr 176), 2013.

16. Lin, K., et al., Preclinical Development of an Anti-NaPi2b (SLC34A2) Antibody-Drug Conjugate as a Therapeutic for Non-Small Cell Lung and Ovarian Cancers. Clin Cancer Res, 2015.

17. Howard A. Burris, M.S.G., David E. Gerber, David R. Spigel, David S. Mendelson, Joan H. Schiller, Yulei Wang, Younjeong Choi, Robert S. Kahn, Katie Wood, Daniel J. Maslyar, Jeffrey R. Infante, A phase I study of DNIB0600A, an antibody-drug conjugate (ADC) targeting NaPi2b, in patients (pts) with non-small cell lung cancer (NSCLC) or platinum-resistant ovarian cancer (OC). J Clin Oncol, 2014.

18. AMG 172 First in Human Study in Patients With Kidney Cancer. NCT01497821, 2015.

19. Taofeek Kunle Owonikoko, A.H., Walter Michael Stadler, David C. Smith, Mario Sznol, Ana M. Molina, Parul Gulati, Aadhar Shah, Christoph Matthias Ahlers, Josephine Cardarelli, Lewis J. Cohen, A phase 1 multicenter open-label dose-escalation study of BMS-936561 (MDX-1203) in clear cell renal cell carcinoma (ccRCC) and B-cell non Hodgkin lymphoma (B-NHL). J Clin Oncol 32:5s, 2014 (suppl; abstr 2558), 2014.

20. John A. Thompson, A.F., Elisabeth I. Heath, Sumanta Kumar Pal, Stephen Maxted Ansell, Jeffrey R. Infante, Sven De Vos, Paul A. Hamlin, Nancy Cherry Whiting, Baiteng Zhao, Nizar M. Tannir; SGN-75 in the treatment of patients with CD70-positive malignancies including metastatic renal cell carcinoma. J Clin Oncol 31, 2013 (suppl 6; abstr 368).

21. Petrul, H.M., et al., Therapeutic mechanism and efficacy of the antibody-drug conjugate BAY 79-4620 targeting human carbonic anhydrase 9. Mol Cancer Ther, 2012. **11**(2): p. 340-9.

22. A Study Of PF-06263507 In Patients With Advanced Solid Tumors. NCT01891669, 2015.

23. ASG-15ME is a Study of Escalating Doses of AGS15E Given as Monotherapy in Subjects With Metastatic Urothelial Cancer. NCT01963052, 2015.

24. SC16LD6.5 in Recurrent Small Cell Lung Cancer. NCT01901653, 2015.

25. Ulrik Niels Lassen, D.S.H., Nikolaos Diamantis, Vivek Subbiah, Rajiv Kumar, Morten Sorensen, Steen Lisby, Robert L. Coleman, Johann Sebastian De Bono, A phase I, first-in-human study to evaluate the tolerability, pharmacokinetics and preliminary efficacy of HuMax-tissue factor-ADC (TF-ADC) in patients with solid tumors. J Clin Oncol 33, 2015 2015.

26. Sussman, D., et al., SGN-LIV1A: a novel antibody-drug conjugate targeting LIV-1 for the treatment of metastatic breast cancer. Mol Cancer Ther, 2014. **13**(12): p. 2991-3000.

27. PCA062 in pCAD-positive Tumors. NCT02375958, 2015.

28. Galsky, M.D., et al., Phase I trial of the prostate-specific membrane antigen-directed immunoconjugate MLN2704 in patients with progressive metastatic castration-resistant prostate cancer. J Clin Oncol, 2008. **26**(13): p. 2147-54.

29. DiPippo, V.A., et al., Efficacy studies of an antibody-drug conjugate PSMA-ADC in patient-derived prostate cancer xenografts. Prostate, 2015. **75**(3): p. 303-13.

30. Hemmerle, T., et al., The antibody-based targeted delivery of TNF in combination with doxorubicin eradicates sarcomas in mice and confers protective immunity. Br J Cancer, 2013. **109**(5): p. 1206-13.

31. A Study of DEDN6526A in Patients With Metastatic or Unresectable Melanoma. NCT01522664, 2015.

32. Xiangbao, Y., et al., Humanized anti-VEGFR-2 ScFv-As2O3-stealth nanoparticles, an antibody conjugate with potent and selective anti-hepatocellular carcinoma activity. Biomed Pharmacother, 2014. **68**(5): p. 597-602.

33. Brack, S.S., et al., Tumor-targeting properties of novel antibodies specific to the large isoform of tenascin-C. Clin Cancer Res, 2006. **12**(10): p. 3200-8.

34. doi: 10.1158/1538-7445.AM2014-2641 Cancer Res October 1.

35. Zhu, M., et al., Neutralizing monoclonal antibody to periostin inhibits ovarian tumor growth and metastasis. Mol Cancer Ther, 2011. **10**(8): p. 1500-8.

36. Lambert, J.M. and R.V. Chari, Ado-trastuzumab Emtansine (T-DM1): an antibody-drug conjugate (ADC) for HER2-positive breast cancer. J Med Chem, 2014. **57**(16): p. 6949-64.

37. doi: 10.1158/1538-7445.AM2015-639 Cancer Res August 1.

38. Elgersma, R.C., et al., Design, Synthesis, and Evaluation of Linker-Duocarmycin Payloads: Toward Selection of HER2-Targeting Antibody-Drug Conjugate SYD985. Mol Pharm, 2015. **12**(6): p. 1813-35.

39. Phillips, A.C.B., E.R.; Vaidya, K.S.; Ansell, P.J.; Shalinsky, D.R.; Zhang, Y.; Voorbach, M.J.; Mudd, S.; Holen, K.D.; Humerickhouse, R.A.; et al, . ABT-414: An Anti-EGFR Antibody-Drug Conjugate as a Potential Therapeutic for the Treatment of Patients with Squamous Cell Tumors. In Proceedings of the 25th EORTC-NCI-AACR Symposium on Molecular Targets Cancer Therapeutics; Boston, MA, USA: , 19-23 October 2013.

40. doi: 10.1158/1538-7445.AM2014-4513 Cancer Res October 1.

41. Hamblett, K.J., et al., AMG 595, an Anti-EGFRvIII Antibody-Drug Conjugate, Induces Potent Antitumor Activity against EGFRvIII-Expressing Glioblastoma. Mol Cancer Ther, 2015. **14**(7): p. 1614-24.

42. Ansell, S.M., Brentuximab vedotin. Blood, 2014. **124**(22): p. 3197-200.

43. Cardarelli, P.M., et al., In vitro and in vivo characterization of MDX-1401 for therapy of malignant lymphoma. Clin Cancer Res, 2009. **15**(10): p. 3376-83.

44. Ohanian, M., et al., Inotuzumab ozogamicin in B-cell acute lymphoblastic leukemias and non-Hodgkin's lymphomas. Expert Opin Biol Ther, 2015. **15**(4): p. 601-11.

45. Pfeifer, M., et al., Anti-CD22 and anti-CD79B antibody drug conjugates are active in different molecular diffuse large B-cell lymphoma subtypes. Leukemia, 2015. **29**(7): p. 1578-86.

46. Chevallier, P., et al., Vincristine, dexamethasone and epratuzumab for older relapsed/refractory CD22+ B-acute lymphoblastic leukemia patients: a phase II study. Haematologica, 2015. **100**(4): p. e128-31.

47. Palanca-Wessels, M.C., et al., Safety and activity of the anti-CD79B antibody-drug conjugate polatuzumab vedotin in relapsed or refractory B-cell non-Hodgkin lymphoma and chronic lymphocytic leukaemia: a phase 1 study. Lancet Oncol, 2015. **16**(6): p. 704-15.

48. Hong, E.E., et al., Design of Coltuximab Ravtansine, a CD19-Targeting Antibody-Drug Conjugate (ADC) for the Treatment of B-Cell Malignancies: Structure-Activity Relationships and Preclinical Evaluation. Mol Pharm, 2015. **12**(6): p. 1703-16.

49. Ribrag, V., et al., A dose-escalation study of SAR3419, an anti-CD19 antibody maytansinoid conjugate, administered by intravenous infusion once weekly in patients with relapsed/refractory B-cell non-Hodgkin lymphoma. Clin Cancer Res, 2014. **20**(1): p. 213-20.

50. Kelly KR, C.-K.A., Somlo G, et al: Indatuximab ravtansine (BT062) in combination with lenalidomide and low-dose dexamethasone in patients with relapsed and/or refractory multiple myeloma: Clinical activity in len/dex-refractory patients. 2013 ASH Annual Meeting. Abstract 758. Presented December 9, 2013.

51. Govindan, S.V., et al., Milatuzumab-SN-38 conjugates for the treatment of CD74+ cancers. Mol Cancer Ther, 2013. **12**(6): p. 968-78.

52. Anastasios Stathis, K.J.M., Ian Flinn, Alex Mejia, Maria Lia Palomba, Sybil Zildjian, Mary Murphy, Jutta Deckert, Rodrigo Ruiz-Soto, Arnold Freedman;, Preliminary findings from a phase I, multicenter, open-label study of the anti-CD37 antibody-drug conjugate (ADC), IMGN529, in adult patients with relapsed or refractory non-Hodgkin lymphoma (NHL). J Clin Oncol 2014. **32:5s,**.

53. Kathleen Whiteman, H.J., Alan Wilhelm, Michael Miller, Wei Li, Emily Reid, Katie Archer, Nathan Fishkin, Andre Dandeneau, Erin Maloney, Jan Pinkas, and Ravi Chari, Abstract C162: Antibody-Drug Conjugates (ADCs) with novel IGN DNA-alkylating agents display potent antigen-specific activity against hematologic and solid tumor xenograft models. Mol Cancer Ther November 2013 12;.

54. Kung Sutherland, M.S., et al., SGN-CD33A: a novel CD33-targeting antibody-drug conjugate using a pyrrolobenzodiazepine dimer is active in models of drug-resistant AML. Blood, 2013. **122**(8): p. 1455-63.

55. Tina M. Albertson, L.S., Baiteng Zhao, Ana Kostic, and Che-Leung Law, Abstract DDT01-04: SGN-CD19A: A novel Anti-CD19 antibody drug conjugate. Cancer Res, 2014. **Cancer Res October 1, 2014 74; DDT01-04**.

56. A Phase I Study of IGN523 in Subjects With Relapsed or Refractory AML. NCT02040506, 2015.
